# Perception of HIV/AIDS among the Igbo of Anambra State, Nigeria

**DOI:** 10.1080/17290376.2013.807052

**Published:** 2013-06-28

**Authors:** Caroline Okumdi Muoghalu, Samuel Ayodele Jegede

**Keywords:** perception, disease, epidemic, knowledge, PLHIV, HIV/AIDS and stigmatization, La perception, La maladie, L'épidemie La connaissance, GVVIH, Le VIH/SIDA et La stigmatisation

## Abstract

Perception is fundamental in the fight against stigmatization of people living with HIV/AIDS (PLHIV). Perception generally influences discriminatory attitudes towards PLHIV which exacerbates their problems and quickens the degeneration of the disease from HIV to AIDS. This study examined the Anambra people's perception and knowledge of HIV/AIDS with the goal of creating knowledge on these issues in order to design effective intervention programmes towards the reduction of social stigmatization associated with the pandemic. The study was carried out in Idemmili North and Oyi local government areas of Anambra State. Qualitative and quantitative methodologies were used to elicit information from respondents who were adult males and females of 18 years and above. The research instruments were questionnaires and in-depth interview schedule. Questionnaires were administered on 1000 respondents while 13 people were interviewed in-depth. Analysis of quantitative data were conducted by using the Statistical package for Social Sciences. Univariate analysis in the form of frequencies were conducted which generated the distribution of respondents across the research variables. Furthermore, multivariate analysis were conducted to test the hypotheses and sought for relationships among variables. The qualitative data were reported in themes based on the research objectives and were analysed jointly with the quantitative data. The findings were that majority of the respondents viewed HIV/AIDS as a disease that afflict immoral people and as a punishment from God. Only a handful of them saw the disease as a disease that could afflict anybody. Also, many of the respondents said that AIDS is real but showed a low level of knowledge. It was further indicated that there were significant relationships between educational level, sex, occupation, income influence perception and peoples' reactions to HIV positive status of a relative while there were no significant relationships between these variables and knowledge of HIV/AIDS. It was concluded that these negative perceptions were as a result of the people's low level of knowledge and cultural belief systems, which see a strange illness as punishment from God for disobedience. Furthermore, the fact that most of the socio-economic characteristics of the respondents had significant relationship with perception and reaction to HIV was an indication that most people in the study area had a uniform perception. It was also an indication that government HIV/AIDS awareness programmes were not effective. It was recommended that strategies for effective HIV educational programme should be sought and carried out in the study area. Effective intervention programme have the power to change behaviours and would likely change the people's negative perception and low level of knowledge of HIV/AIDS, thereby reducing stigmatization of people living with HIV/AIDS.

## Abstract (FR)

La perception est indispensable dans la lutte contre la stigmatisation des gens qui vivent avec le VIH/SIDA. (GVVIH) La perception influence d'habitude des comportements discrimatoires envers les gens qui vivent avec le VIH/SIDA ce qui aggrave leurs problèmes et facilite une détérioration de leur santé. On veut examìner le point de vue des gens d'Anambra et leur connnaissance du VIH/SIDA dans le but de mettre en place des programmes d'intervention efficace afin de minimiser la stigmatisation sociale associée à cette pandémie. Le Nord de Idemmili et l'administration communale de Oyi dans l'état d'Anambra ont fait I'objet de notre recherche. Les méthodologies quantitatives et qualitatives ont été employées pour tirer des informations des sondés adultes hommes et femmes de plus de 18 ans. Les instruments de recherche sont les questionnaires et une interview approndie. On a distribué des questionnaires à 1000 sondés tandisque 13 sondés sont passés par une interview approfondie. Une analyse quantitive de données a éte effectuée à l'aide des données statistiques des sciences sociales. Une analyse en forme de fréquences a été effectuée. Beaucoup d'autres analyses ont vu le jour dans le but de tirer des hypothèses et de chercher des rapports entres les variables. Les données qualitatives sont en forme de thème basées sur les objectifs de la recherche et sont conjointement analysées avec les données quantitatives. On se rend compte que la majorité des sondés considèrent le VIH/SIDA comme une maladie des personnes immorales et comme une punition venant de Dieu. Toutefois, une poignée pense que tout le monde peut être victime. Plusieurs sondés ont dit que le SIDA est réel. Cependant ils manquent de connaissance adéquate. On a en plus remarqué un rapport très important entre le niveau de l'éducation, sexe, la profession, le salaire et leur réaction contre un membre de famille qui est séropositif cependant il n'ya Presque pas de rapport entre ces variables et l'image qu'ils ont du VIH/SIDA. On a donc conclu que cette perception negative est causée par une compréhension floue et la croyance culturelle qui considère de punition divine une maladie étrange. On a remarqué un rapport très important entre les caracteristiques socioéconomiques des sondés, leur perception et réaction contre le VIH, ceci indique une perception uniforme du VIH de gens de cette région. C'est aussi la preuve palpable que les programmes de prise de conscience du VIH/SIDA entrepris par le gouvernement manque d'efficacité. On a donc suggeré que les strategies du programme d'éducation effectif contre le VIH soit encouragé dans la région. Un programme d'intervention efficace peut changer les comportements et pourrait changer la perception négative de gens et la connaissance floue du VIH/SIDA ce qui minimiserait la stigmatisation de gens qui vivent avec le VIH/SIDA.

## Introduction

The perception of HIV/AIDS has been a big problem owing to the its central place in the discrimination, stigmatization and attitudes towards people living with HIV/AIDS (PLHIV) throughout the world. Sabatier (1988) noted that due to the initial misconceptions and negative reaction to the disease, people affected with HIV/AIDS have been blamed, stigmatized and isolated. The HIV/AIDS pandemic has been topical since the identification of the disease in the early 1980s. Indeed, it is a major event of our time. It has killed over 80,000 people and 70% of these were in sub-Saharan Africa (United Nations Population Division 2003). UNAIDS and WHO (2008) global epidemic update indicated that 33.4 million people were infected and that 2.7 million were newly infected in 2008. Also in 2008, total AIDS-related deaths was 2.0 million people. Sub-Saharan Africa has the world's highest HIV prevalence and as a result faces the greatest demographic transition. Of the 33.4 million PLHIV worldwide, 70% are in sub-Saharan Africa (UNAIDS & WHO 2008).

A 2004 report from UNAIDS projected that more than 80 million Africans could die from AIDS and infections could soar to 90 million or more than 10% of the continents population. Presently, Africa accounts for 90% of infected babies in the world. The more number of women living with HIV/AIDS in Africa is a result of certain cultural practices and the unequal gender relations, which render women and girls vulnerable to STDs including HIV/AIDS. At the onset of the HIV/AIDS pandemic, more males were infected but the trend has been reversed due to these cultural practices (UNAIDS 2006).

According to UNAIDS (2006), 300,000 adults were newly infected in 2005 making it a total of about 2.9 million within the range of 1.7 million and 4.2 million PLHIV in Nigeria. By the end of 2009, the HIV seropositive rate was 3.1% with a number of women as many as 1.4 million, and children – 220,000 and death due to AIDS in 2009 in Nigeria were 170, 000 (UNAIDS 2009). Also, UNAIDS (2009) had it that 2.6 million Nigerians are living with HIV/AIDS. The HIV/AIDS epidemic in Nigeria shows a lot of variation. The statewide prevalence ranges from as high as 10% in Benue and 8% in Akwa Ibom to under 2% in Ekiti, Oyo and Jigawa (Federal Ministry of Health 2006). In Nigeria, males have also been shown to be the main bridging route between people who engage in high-risk sexual behaviour such as female sex workers and the general population (Adeyi, Kanki, Odutolu & Idoko 2006).

Low level of knowledge of the disease has also been indicated as one of the problems associated with HIV perception and misconceptions. In a study of Barbing saloon operators in Sokoto, Nigeria, Ibrahim, Opara and Tanimomo (2007) found that there is a very low level of knowledge of the risk of transmission of HIV infection through unsterilized equipment in barbing saloons. Chng, Eke-Huber, Eaddy and Collins (2005) in a study of perception of HIV/AIDS in selected Tertiary institutions in Southern Nigeria found that many of the respondents believed that antibiotics can cure HIV/AIDS. In the same vein, Kaiser Family Foundation (2006) found that there were misconceptions about HIV transmission in all segments of American population in which 37% believed that it might be transmitted through kissing, 22% through sharing a drinking glass, 16% through touching a toilet seat and that at least 43% adult population hold at least one of these misconceptions. This low level of knowledge of the disease is a big factor fuelling the problem of stigmatization which quickens the death of PLHIV. This stigma is also a product of the perception of the disease. The perception of the disease influences to a great extent the people's attitude to PLHIV. This has made the perception of HIV/AIDS a very important aspect of the HIV/AIDS crises with far reaching effects.

Owing to the fact that HIV/AIDS is to some extent a behavioural disease, PLHIV have been perceived by many as immoral people (Caldwell, Caldwell, Anarfi, Awusabo-Asare, Ntozi, Orubuloye, *et al.* 1999). Caldwell *et al.* (1999) captured this in their report when they said that people prefer not to talk about AIDS partly because it was an unusual disease with mysterious symptoms perhaps related to the occult and partly because it was associated with sexual behaviour in a society that finds it difficult to talk about sex publicly and across generational boundaries.

It is this perception of the disease that influences the attitude of the public to PLHIV. For instance, UNAIDS (2003) described the Nigerian public as a people who know little about HIV/AIDS and who discriminate against PLHIV with little or no sympathy. Despite the disease's impact in Nigeria, the historical lack of both private and public efforts around disease prevention has resulted in a public that is largely uninformed and unconcerned about the epidemic. In addition, the high levels of stigma and discrimination have stifled personal investment in the disease (UNAIDS 2003).

In the same vein, Alubo, Zwandor, Jolayemi and Omudu (2002) in a study of stigmatization of PLHIV in Benue State found that at the community level, AIDS was perceived as a ‘just reward’ for immorality, according to them, ‘the mouth that eats pepper feels the bitterness’. In spite of the fact that the whole family was often labelled ‘AIDS family’, the children of the PLHIV were often taunted as having AIDS mama or papa; there was thus the possibility that these reactions might jeopardize marriages and other future relations. As a reflection of low acceptance and concomitant stigma, the funeral rites of PLHIV were different. They were not laid in state and the usual 7–14 days mourning period for normal deaths was not observed, people disperse immediately after burial. There were also suggestions in the community that the grave for AIDS deaths should be much deeper to guard against any possibility of post-burial infection. In the same study, the family members of PLHIV indicated that the level of isolation and rejection by the public was too high.

AIDS in a family member sometimes lead to tension between the family and neighbours with the latter reducing their contact. Some people living with AIDS withdraw socially, giving up activities that bring them into contact with non-relations such as attending church and village meetings. Anarfi (1992) found that researchers were sometimes treated like neighbours and family refused access to PLHIV. Anarfi also reported that in Ghana, there were cases of people living with AIDS being shut away in one room because of the negative perception and stigmatization. Awusabo-Asare (1995) reported of a young man who returned from studies abroad and found that he was HIV positive; he preferred to stay in hospital till death. This young man did this due to fear of a negative perception and discrimination even by family members. This could be because AIDS as a disease has no cure and it is interpreted as a curse or punishment for disobedience. Such a situation brought shame not only to the individual but also to the corporate clan (Bleek 1981). In the same vein, Berer and Ray (1993) corroborated this finding in an opinion poll in Morocco, which found that people taught that one measure was essential for stopping the spread of HIV/AIDS – the imprisonment of all sex workers.

There is general inaction on the part of the government, individuals, community and other groups. Malungo (1998) observed that no one blamed the government for inaction, even in countries where over one-quarter of adults are currently seropositive and where most of the population will die of AIDS. This general perception has portrayed PLHIV as untouchables and this has equally raised the level of stigma. Even religious bodies are not left out of this perception. A study of South Western Nigeria by Olubuloye, Caldwell and Caldwell (1993) found that three-quarters of Christian and Moslem clergy in Nigeria believed that AIDS was a divine punishment from God and the proportion was not likely to be lower in the East and Southern Nigeria or among the laity of the congregations. In the same vein, Aniebue (2006) also found that the Nigerian clergy still attributed HIV/AIDS to a divine punishment from God and to the activities of demonic and spiritual forces. These misconceptions had also been part of the problem of HVI/AIDS in the sense that it had serious implications for preventive efforts and care for PLHIV.

Another problem of these misconceptions was that many people did not view themselves as being at risk of contracting HIV. In a study of knowledge, perception and acceptability of microbicides among non-healthcare workers in Lagos, Nigeria, Smith, Adeiga and Agomo (2008) found that those at high risk of HIV acquisition, hairdressers and truck drivers' spouses and partners were not willing to use the microbicide because they did not see any need for it as they did not see how contracting HIV concerns them. Also, Odu and Akanle (2008) in a study of youth in South Western Nigeria found that there had been misconceptions; that most of the youth engaged in high-risk sexual behaviour with the belief that antibiotics can cure the disease.

According to UNAIDS and World Health Organization (2001), healthcare sector has generally been the most conspicuous context for HIV-related discrimination. Negative attitudes from healthcare staff have generated anxiety among PLHIV. As a result, many kept their status secret. Indeed, there was almost hysterical kind of fear at all levels starting from the humblest, the sweeper or the ward boy, up to the heads of departments which made them pathologically scared of having to deal with an HIV positive patient. There were also examples of discriminations of children of HIV positive parents whether positive or negative themselves. Such children were being denied the right to go to school and they were being separated from other children. This was corroborated by UNAIDS (2004) epidemic update research in four Nigerian states which found discriminatory and unethical AIDS-related behaviour among doctors, nurses and midwives – denial of care, testing without consent, and breaches of confidentiality. One in 10 care providers reported refusing to care for HIV positive patients, 10% reported refusing them admission to a hospital. Furthermore, 65% reported seeing other healthcare workers refusing to care for an HIV or AIDS patient. Some 20% felt that many people living with HIV had behaved immorally and deserved to be infected.

This perception was influenced by a lack of sympathy for the other, lack of knowledge about the disease and traditional belief systems. Traditional society believes that non-marital sex should be surreptitious and that public revelation or being caught out often resulted in punishing illnesses and other disasters. Bleek (1981) noted that this can be associated with an older sense of shame, that is, fear of bringing disaster to the community because suffering from an unusual or previously unknown disease can be related to sin which must have been committed by the infected individual but which would, nevertheless, bring punishment on the whole society. One of the reactions of relatives when this occurs is to distance themselves from the shame and ridicule brought by a member of the family or corporate clan. Bleek (1981), therefore, maintained that it is within this context that the reaction of some people to HIV/AIDS infection should be viewed. Such interpretation obviously influences perception of a particular disease, people living with it and the behaviour of infected persons. The shame of being HIV positive was pronounced throughout the interview with people living with AIDS and their families (Bleek 1981). It is within all these beliefs, conceptions and misconceptions that people's perceptions of HIV/AIDS are located.

This study examined Anambra people's knowledge and perceptions of HIV/AIDS by exploring the following objectives:

- To examine peoples' knowledge of HIV/AIDS
- To explore the respondents' view of HIV/AIDS
- To examine the peoples' reactions to HIV positive relations

## Rationale for the study

Many studies on HIV/AIDS in Nigeria have focused on identification of individual attitudes, stigma, and care and support. Moreover, most studies in Nigeria have focused on high-risk groups – sex workers, male clients of sex workers and intravenous drug users and on biomedical perspectives (hospital care, and health workers) ignoring the link between human action and socio-cultural factors. Under this situation, people's attention could be turned away from critical questions that must be understood only by examining the socio-cultural context of behaviour and this is the gap the present study is addressing. The study is addressing this gap by studying people in their particular society instead of individuals who came to live in towns from different cultural contexts. There is, therefore, need to explore peoples' perception of HIV/AIDS in the socio-cultural context of the Anambra Igbo because it is believed that the socio-cultural context influences perception of HIV/AIDS. Furthermore, knowing their perception as a people would inform the kind of intervention programmes that would be effective in the bid to reduce negative perception and reduce stigma in order to contain the epidemic.

The study focused on Anambra because Anambra State was identified (Family Health International 2001) as one of the expected breeding grounds for HIV infection due to the presence of the commercial town of Onitsha and long distance drivers.

## Social and cultural context for the study

This study was carried out among the Igbo people of Anambra State, Nigeria. The Igbo people live in the east of the River Niger and they speak the Igbo language. They are known for their industry, doggedness, business acumen, hospitality and a strong belief in their cultural practices and their ancestors. They are traditionally farmers and petty traders but some are now civil servants. They farm at the subsistence level and live communally. The Igbos practice African traditional religion but many are now Christians due to the influence of Christianity and Westernization. Interestingly, this does not stop the people from conducting their traditional festivals and rituals and observing their cultural practices and beliefs and this makes the people to combine Christianity and traditional religion, making it difficult to tell who is a Christian and who is a traditional religious worshipper. The African traditional religion as practised in the area is so pervasive that every activity (ranging from birth, marriage, burial to health-seeking behaviour which starts with divination) is embedded in it. This is why divination is very central to health seeking and healing in the area. Divination is the main means of diagnoses. However, in recent times, divination is combined with hospital care. The welfare system in the study area is mainly the communal life led by community members; supporting one another when they are in need. There is no government structure or policy with relevance to PLHIV in the study area.

Politically, Anambra people traditionally do not have a centralized system of government. The power is in the hands of the elders who are also the kingmakers and who take the opinion of the majority into consideration before taking decisions. Kings are chosen by the people and they work for the benefit of the people at community level and their power is moderated by the elders. One can describe their style of government as republican. However, at the state level, there is a state governor who represents the state at the federal level. Many communities in Anambra State are rural with the exception of few towns that can be called urban. The socio-cultural context of Anambra state is akin to what is obtained in many parts of Nigeria, West Africa and other sub-Saharan African countries in the sense that there is hardly any place in sub-Saharan Africa that one cannot find the kind of economic, political and religious arrangement practised by Anambra people. However, Anambra is distinct in the sense that their strong attachment to their cultural and religious beliefs is so pronounced that it influences their every activity, including their perception of HIV/AIDS.

## Conceptual framework

In this study, perception of HIV/AIDS was conceived as a product of the traditional belief systems of the people and the definitions of the situation of the people as informed by their cultural and environmental factors and level of knowledge.

## Perception

Perception is the process by which organisms interpret and organize sensation to produce a meaningful experience of the world (Lindsay & Norman 1977). Perception in this study means the way the people view and define HIV/AIDS and people living with the disease.

## Theoretical framework

The theoretical framework for this study was the action frame of reference (Max Weber 1960; Parsons 1937), which posited that the action of an individual towards an issue or object is determined or influenced by the definition of the situation. This sociological perspective focuses on the acting individual and the acting group. The action frame of reference gives a complete and comprehensive picture of the interaction between the actor and the environment in which the actor operates. As explained by Weber (1960), explanation of social action must arise from the definitions of the situation and purposes of the actors. In action is included all human behaviour when and insofar as the acting individual attaches a subjective meaning to it. Action in this sense may be overt or purely inward or subjective.

Weber postulated that cultural values circumscribe and direct social action and as such, another main defining agency is the community. Owing to Weber's overt emphasis on individual meaning, Talcott Parsons Voluntaristic Social Action Theory was employed to strengthen the shared meaning aspect of human action, which is the main force in individual behaviour. Parson's Voluntaristic Action Theory is a variant of the functionalist perspective. This theory emphasizes constraint of individual within particular customs and values. This helps to explain human behaviour with regard to socio-cultural factors and their influence on perceptions and attitudes. Much like Weber's action theory, which asserts the primacy of the society over the individual (Giddens 2000), it argues that societies exert social constraint over the actions of individuals.

This perspective focuses on the course of action as determined by the conditions of the cultural, physical and social environment; society influences the end which the actor seeks and the means he/she will use in attaining them. Parsons' theory like that of Weber states that action can be explained in the context of the subjective meaning, given to it by the actor and that actions are always directed at the attainment of goals with the choice of the most appropriate method by the actors.

Applying action theory to the study of perception of Anambra people to HIV/AIDS and PLHIV, the assumption is that social action (in this case; perceptions) must arise from the definition of the situation, which is to a great extent culturally defined. That is to say that how the people define (perceive) the HIV/AIDS disease and people living with it is determined by the cultural beliefs of the people. This definition of the situation also influences the behaviour of PLHIV. It is this culture's ability to define the situation for the person that is responsible for these uniformities in behaviour of a particular people (in this case Anambra people's perception of HIV/AIDS).

### Statement of hypothesis

Hypothesis 1

H_0_: There is no significant relationship between socioeco- nomic characteristics of respondents and knowledge of HIV/AIDS.

Hypothesis 2

H_0_: There is no significant relationship between socioeco- nomic characteristics of respondents and perception of HIV/AIDS.

Hypothesis 3

H_0_: There is no significant relationship between socioeco- nomic characteristics of respondents and reaction if a relative tests positive for HIV/AIDS.

## Methodology

The study employed qualitative and quantitative data collection methods to generate data for the examination of perceptions of HIV/AIDS in the study area. The independent variables are age, sex, educational level and marital status. In order to get a deep insight into the study problem, in-depth interviews were conducted to complement data from the survey. The combined use of these methods strengthened the result of the research because the weakness of one method was strengthened by the strength of the other.

In selecting respondents for the administration of questionnaires, Household census was carried out in three purposively selected communities in each local government area (Umuoji, Ogidi and Nkpor in Idemmili North and Nteje, Ogbunike and Nkwele- Ezunaka in Oyi). These communities are the three biggest communities in each of the selected local government and as such are the microcosm of the Local Government Areas. All the households in each selected community were listed serially to form the sampling frame. Systematic random technique was used in selecting every fifth household. The systematic random method has the strength of making the sample more representative of the population. In the selected households, questionnaires were administered on adult males and females (18 years and above). In each selected household, there was balloting (if there were more than one adult in a household, the researcher would bring pieces of paper and writes ‘no’ in two and ‘yes’ in one) and any of the adults in the household that picks yes was given the questionnaire. This means that only one respondent was chosen from each selected household.

Four university graduates of the social sciences were recruited and trained as research assistants for the data collection. A total of 1000 questionnaires were administered on 1000 respondents and 914 were completed successfully and analysed. The qualitative methods for the study (in-depth interviews) were conducted on 6 caregivers and 6 opinion leaders (12 people). Participation in the interviews was voluntary.

The data collection was carried out between February 2009 and July 2009. Before the commencement of the data collection, a short proposal was written and submitted to the ethical committee of the University of Ibadan/University College Hospital, Ibadan which duly approved the research project. Also efforts at informed consent were made by contacting the community leaders and having a series of meetings with them. During the meetings, the nature and purpose of the research were explained to them. They were also assured that all information gathered during the data collection would be kept confidential and that no one would come to any harm because of it. The community leaders then called a meeting of all the villagers and introduced the research team to them and assured them of confidentiality of their opinions and their safety. The research team then used this opportunity to seek the consent of the villagers and explained every thing about the research to them. The villagers unanimously agreed to participate in the research and this paved the way for a hitch-free research. Before the commencement of the research proper, a pilot qualitative study was carried out in January 2009 administering 40 questionnaires and conducting four interviews to elicit information from respondents based on the objectives of the research. The result of this study helped in designing the questionnaires and the interview guide. The questions in the questionnaires and interview guide fill the gap in the previous research on the subject, in the sense that the previous research on the subject was mainly on the individual level of perception, ignoring the socio-cultural context of individual behaviour.

For the in-depth interviews, the members of the communities identified opinion leaders (three females and three males) in their communities. Opinion leaders are men and women of integrity in the villages. The people have great respect for them and anything they say is taken seriously without any argument from the people. Achieving the status of an opinion leader does not happen in one day. Opinion leaders have built their reputation over time through good deeds, honesty, hard work and they handle community projects as if it is their personal project and by rendering help to members of the community. They have, therefore, through these means endeared themselves to the members of the community. They are, therefore, trusted and respected. The cultural significance of opinion leaders is that their opinion represents the opinion of the people. As such, opinion leaders by virtue of their position can conveniently tell the opinion of the people because they have constant and daily contact with the people and they hear and know what the people need or can say on any issue. Also the people trust them and can tell them their inner most feelings. The opinion leaders are, therefore, the mouthpiece of the people. The opinion leaders were reached in their family homes and the participation was voluntary.

Qualitative data generated through In-depth interviews were analysed using ethnographic software for qualitative data analysis version 0.5. Responses were arranged thematically based on the objectives. Analyses of quantitative data were both descriptive and inferential, using SPSS software version 11.1 to enter the data in the system. Univariate analysis in the form of frequencies and percentages contributed to understanding of the distribution of each variable across survey respondents. Multivariate analyses were conducted using a one-way analysis of variance (ANOVA) statistics to test for categorical factors and to establish relationship between dependent and independent variables. In the end, both quantitative and qualitative data were analysed jointly by using the qualitative data to support findings from the quantitative data.

## Justification for using systematic sampling method:

Systematic sampling method was used because it has the strength of making the sample more representative of the population. By choosing every fifth household, it makes it possible for the sample to spread out so that more areas were covered which helped to include more households instead of choosing only people who live very close to one another. Using the systematic sampling technique made it possible to give every household equal opportunity to be selected in the sampling frame.

## Techniques employed to remove error/bias

In order to minimize bias/errors, objective sampling technique was used. Systematic sampling technique is a random sampling technique which minimizes bias/errors in the conduct of research. This made it possible for every household in the study area to have equal chance of being selected and during the final selection of the participants, balloting was carried out within the household which also gave every adult member of the family an equal chance of being selected. Also, before the commencement of the study proper, a pilot qualitative study was carried out in January 2009 in which eight in-depth interviews were conducted to elicit information from respondents based on the objectives of the study. The result of this study helped in designing the questionnaires and the interview guide. The interview questions for the pilot study originated from the literature reviews on HIV/AIDS care in Nigeria/Africa. Furthermore, in the recruitment of the research assistants, it was ensured that the four of them were social science graduates who had previously conducted field study in social sciences and had acquired experience in this regard. Apart from this, the research assistants were trained for the purpose of this study using the research instruments as a training manual. This was to ensure uniformity in the data collected. Also, some of the participants were visited a number of times to retrieve the questionnaires. This was in order to reduce the attrition rate.

## Age categories in Table 1

These age categories were just used to describe the age distribution of the respondents. I am only interested in description and inferential statistics and not the dispersal analysis of the respondents.

**Table 1.** Socio-demographic characteristics of respondents.

Variables	Frequency	%
Age (years)		
18–27	148	16.8
28–37	563	61.6
38–47	159	17.4
48 and above	44	4.8
**Total**	**914**	**100**
*Marital status*		
Married	657	71.9
Single	216	23.6
Divorced	17	1.9
Separated	9	1
Widowed	15	1.6
**Total**	**914**	**100**
*Educational level*		
No education	70	7.7
Primary	205	22.7
Secondary	512	56.0
Tertiary	127	13.9
**Total**	**914**	**100**
Sex		
Male	461	50.4
Female	453	49.6
**Total**	**914**	**100**
*Religion*		
Christianity	879	96.2
Islam	4	0.4
Traditional religion	31	3.4
**Total**	**914**	**100**
*Occupation*		
Trading	322	35.2
Civil service	252	27.6
Artisanship	111	12.1
Blue-collar jobs	68	7.5
Students	50	5.5
Unemployed	111	12.1
**Total**	**914**	**100**

*Source:* Author's field survey (2009).

## Findings

This table showed the social, economic and demographic features of the respondents, using the following variables: age, marital status, educational level, religion, occupation and sex. Table 1 showed that 16.8% of the respondents were within the ages of 18–27 years, while 61.6% were within the ages of 28–37 years, 17.4% were 38–47 years and 4.8% were 48–60 years. The table also showed that 7.7% had no formal education, 22.4% had primary education, 56.0% had secondary education while 13.9 had tertiary education. The table showed that majority of the respondents were secondary school graduates, which was an indication of the fact that people in the study area were not highly educated. These aspects have implications for the people's level of knowledge of HIV/AIDS and their perception of the disease.

Table 1 further indicated that 71.9% of the respondents were married, 23.6% were single, 1.9% were divorced, 1% were separated and 1.6% were widowed. Moreover, according to the table, 96.2% were Christians, 0.4% were Muslims while 3.4% practice traditional religion. Table 1 also indicated that 35.2% were traders, 27.6% were civil servants, 12.1% were artisans, 7.5% were in blue-collar jobs, 5.5% were students and 12.1% were unemployed.

The people's knowledge of HIV/AIDS was explored because it was believed that their knowledge of HIV/AIDS influenced their perception of HIV/AIDS.

Table 2 indicated that 99.4% of the respondents have heard about HIV/AIDS while the rest have not heard of it. Also 27.2% of the respondents said that they heard it from a friend, 71.8% heard it from radio and television, 13.2% heard it from the market. Other respondents, 2.7% heard it from church, 2.7% from hospital, 1.9% from the newspaper, 3.7% from school and 1.0% from office. Furthermore, the majority (98.2%) of the respondents agreed that AIDS is real. On how it is contracted, 92.0% said that it is contracted through sexual intercourse while, 11.5% indicated that it is contracted through hospital, and 1.8% through shaking of hands. Meanwhile, 22% of the respondents indicated that they use condoms in order to protect themselves, 51.0% abstain from sex, 3.0% use medicine and 24.0% do nothing to protect themselves. The responses in Table 2 showed that most of the respondents have heard about HIV/AIDS. They knew some of the sources through which it could be contracted and the majority indicated that everybody was at risk. However, there were still others who showed a low level of knowledge of the disease by indicating that they used medicine to protect themselves against the disease. There were also those that said that AIDS is contracted through shaking of hands.

**Table 2.** Distribution of knowledge of HIV/AIDS among the respondents.

	Frequency (*n* = 914)	% (100%)
*Ever heard of HIV/AIDS*		
Yes	900	99.4
No	14	0.6
*From whom did you hear it?*		
Friend		
Yes	249	27.2
No	651	72.8
Radio and television		
Yes	656	71.8
No	258	28.2
Market		
Yes	121	13.2
No	793	86.8
Other sources of information		
Church	25	2.7
Hospital	25	2.7
Newspaper	17	1.9
School	34	3.7
Office	9	1.0
*Is AIDS real?*		
Yes	889	98.2
No	25	1.8
*Modes of transmission*		
Sex		
Yes	842	92.0
No	72	8.0
Through hospital		
Yes	109	11.5
No	805	88.5
Through shaking of hand		
Yes	20	1.5
No	894	98.5
*Is everybody at risk?*		
Yes	740	81.8
No	174	18.2
*What have you done to protect yourself?*		
Condom		
Yes	199	22.0
No	715	78.0
Abstain		
Yes	462	51.0
No	452	49.0
Medicine		
Yes	27	3.0
No	887	97.0
Nothing		
Yes	217	24.0
No	697	76.0

*Source*: Author's field survey (2009).

Furthermore, data from the in-depth interviews showed that many people in the study area did not know much about the disease. For instance, a male informant said that the disease is so strange that they feel that someone can contract it through the air. This great fear of contraction of the infection means that many of the people would not want to come near or have anything to do with PLHIV. This low level of knowledge affects perception and inhibits how PLHIV cope with the disease. Also during an interview session, an opinion leader said that he did not believe that AIDS is real. To him, it was the white man's propaganda to curb men's libido. He maintained that he had not seen anybody who said he/she had AIDS. These responses showed lack of knowledge of the disease which were likely to affect the people's perception.

According to Table 3, 34.3% of the respondents indicated that AIDS afflicts immoral people, 20.6% saw it as caused by a germ, 25.9% saw AIDS as a punishment from God, 4.3% saw it as a disease that can afflicted anybody while 14.9% did not know. The fact that almost 15% of the respondents indicated that they did not know how to define HIV/AIDS was a sign of a low level of knowledge of the disease and could have implications for perception of HIV/AIDS.

**Table 3.** Distribution of respondents by perception of HIV/AIDS.

Variables	Frequency	%
Afflicts immoral people	314	34.3
Natural/germ	188	20.6
God's punishment	237	25.9
Can afflict anybody	39	4.3
Do not know	136	14.9
Total	914	100

*Source*: Author's field survey (2009).

According to Table 4, 54.1% of the respondents would care for PLHIV, while 21.7% would confine them and keep it a secret and 24.2% did not know what they would do in such a situation. Table 4 indicated that a majority of the respondents were ready to care for relations suffering from HIV/AIDS. It is important to note that a family could do more than one of the above for their members living with HIV/AIDS.

**Table 4.** Respondents' reactions to relations' HIV positive status.

Variables	Frequency	%
Care and encouragement to seek treatment	487	54.1
Confine/keep secret	196	21.7
Do not know	218	24.2
Total	901	100

*Source*: Author's field survey (2009).

According to a male informant:

In most cases, the family of PLHIV kept him/her in one room and would be taking care of the person by providing his/her needs as much as possible. They accompany him to the herbalist or hospital and they tell outsiders that the person travelled or that he/she is suffering from typhoid fever or poison. No family ever mentions that their family member is living with HIV/AIDS because the shame would be on the entire family.

A male respondent during one of the interview sessions indicated, ‘If my relation is HIV positive, I can only contribute money for his care but cannot go near him/her because I would not like the sight.’

A female opinion leader also indicated that it is difficult to even come near PLHIV, let alone trying to help them. If my family member is living with HIV/AIDS, I would never mention it to anybody outside the family. This is because it is a big shame for all the members of the family. According to her, it is a big shame for the family that one of their own is suffering from a disease of immoral people meaning that the entire family members are likely to be immoral people.

Furthermore, a mother of a widow living with HIV/AIDS said that her family kept her daughter in one room and they were catering for her needs. They tried as much as possible to take care of her, provide her needs, counsel her and assure her that her children would be taken care of even if she died.

## Bivariate/multivariate analysis

### Test of hypotheses

The essence of tests of hypotheses is to validate the hypotheses that are found to be true and reject those that are found to be false. In any social research, Hypothesis provides explanation for certain facts and the relationships between variables.

### Decision rule for testing the study hypotheses

The decision rule is to accept the null hypothesis (H_0_) if the *F*-value (as in the statistical table) is less than the calculated table value and if the *p*-value is > .05. This will indicate that there is no significant relationship between the variables. On the other hand, null hypothesis (H_0_) would be rejected if the calculated *F*-value is greater than the table value; in this case, the alternative hypothesis (H_1_) would be accepted.

Test of Hypothesis 1: H_0_

‘There is no significant relationship between socio-economic characteristics (such as age, sex, level of education, marital status, religion, and occupation) of respondents and knowledge of HIV/AIDS.’ To test this hypothesis, data collected on socio-economic characteristics of respondents and the knowledge of HIV/AIDS were subjected to One-way Analysis of Variance (ANOVA).

In Table 5, shown is the descriptive analysis of one-way ANOVA on the relationship between socio-economic characteristics of respondents and knowledge of HIV/AIDS in Anambra, Nigeria at *p* ≤ .05 level of significance. As shown in the table, the result of the analysis for marital status revealed that the Mean Squares between Groups and Within Groups were 4.728 and 0.935, respectively (Msbg = 4.728, Mswg = 0.935). These yielded the *F^−^*^val^ of 5.055 and *F*^−tab^ of 19.50 which was significant at *p* < .05 level of significance (*F^−^*^val^ = 5.055, *p* ≤ .05). Therefore, from the statistical analysis below, the null hypothesis (H_0_) was rejected and alternative hypothesis (H_1_) accepted. This meant that there was a significant relationship between marital status and knowledge of HIV/AIDS.

**Table 5.** Descriptive ANOVA on the relationship between socio-demographic characteristics of respondents and knowledge of HIV/AIDS.

Socio-demographic characteristics	Relationship between socio-demographic and economic characteristics knowledge of HIV/AIDS						
		Sum of squares	df	Mean square	*F*^−val^	*F*^−tab^	*p* < .05 Sig.
Marital status	Between group	9.456	2	4.728	5.055	19.50	.007
	Within group	843.627	902	0.935			
Total		853.083	904				
Educational level	Between group	4.897	2	2.448	1.562	19.50	.210
	Within group	1413.622	902	1.567			
Total		1418.519	904				
Sex of respondents	Between group	1.768	2	0.884	3.551	19.50	.029
	Within group	224.482	902	0.249			
Total		226.250	904				
Religion	Between group	0.038	2	0.019	0.137	19.50	.872
	Within group	123.149	902	0.137			
Total		123.187	904				
Occupation	Between group	6.806	2	3.403	1.611	19.50	.200
	Within group	1905.795	902	2.113			
Total		1912.601	904				
Income	Between group	7.675	2	3.837	1.373	19.50	.254
	Within group	2520.495	902	2.794			
Total		2528.170	904				

*Source*: Author's field survey (2009).

Note: Significant at *p* < .05.

Also shown is the descriptive analysis of one-way ANOVA on the relationship between educational level of respondents and knowledge of HIV/AIDS in Anambra, Nigeria at *p* ≤ .05 level of significance. The result of the analysis for highest educational attainment indicated that the Mean Squares between Groups and Within Groups were 2.448 and 1.562, respectively, (Msbg = 2.448, Mswg = 1.562). These yielded the *F*^−val^ of 1.562 and *F*^−tab^ of 19.50 which was not significant at *p* < .05 level of significance (*F*^−val^ = 1.562, *p* > .05). Therefore, from the statistical analysis below, the null hypothesis (H_0_) was accepted. This meant that there was no significant relationship between the educational level and the knowledge of HIV/AIDS.

A one-way ANOVA on the relationshipbetween sex of the respondents and knowledge of HIV/AIDS in Anambra, Nigeria at *p* < .05 level of significance is shown Table 5. The result of the analysis revealed that the Mean Squares between Groups and Within Groups were 0.884 and 0.249, respectively (Msbg = 0.884, Mswg = 0.249). These yielded the *F*^−val^ of 3.551 and *F*^−tab^ of 19.50 which was significant at the *p* < .05 level of significance (*F*^−val^ = 3.551, *p* ≤ .05) in-spite of the fact that the *F*^−tab^ was greater than *F*^−val^, suggesting a weak significant relationship. Therefore, the null hypothesis (H_0_) was rejected and alternative hypothesis (H_1_) accepted. This meant that there was a significant relationship between sex and knowledge of HIV/AIDS.

Furthermore, using ANOVA, Table 5 also indicated that there was no significant relationship between religion, occupation and income of respondents and knowledge of HIV/AIDS as the *F*^−tab^ for these variables were greater than their *F*^−val^ and *p* > .05, which was an indication of no significant relationship between these variables and knowledge of HIV/AIDS.

Test of Hypothesis 2: H_0_

‘There is no significant relationship between socio-economic characteristics (age, sex, level of education, marital status, religion, and occupation) of respondents and perception of HIV/AIDS.’ To test this hypothesis, data collected on socio-economic characteristics of respondents and the perception of HIV/AIDS were subjected to One-way Analysis of Variance (ANOVA).

Table 6 shows the descriptive analysis of one-way ANOVA on the relationship between socio-economic characteristics of respondents and perception of HIV/AIDS in Anambra, Nigeria at *p* < .05 level of significance. As shown in the table, the result of the analysis for marital status shows that the mean Squares between groups and within groups were 5.596 and 0.926, respectively (Msbg = 5.596, Mswg = 0.926). These yielded the *F*^−val^ of 6.044 and *F*^−tab^ of 5.63 which were significant at *p* < .05 level of significance (*F*^−val^ = 5.596, *p* < .05). Therefore, from the statistical analysis below, the null hypothesis (H_0_) was rejected and alternative hypothesis (H_1_) accepted. This meant that there was a significant relationship between marital status and perception of HIV/AIDS.

**Table 6.** Descriptive ANOVA on the relationship between socio-demographic characteristics of respondents and perception of HIV/AIDS ANOVA.

Socio-demographic characteristics	Relationship between socio-demographic characteristics of respondents and perception of HIV/AIDS							
		Sum of squares	df	Mean square	*F*^−val^	*F*^−tab^	*p* < .05 Sig.
Marital status	Between group	22.383	4	5.596	6.044	5.63	.000
	Within group	829.546	896	0.926			
Total		851.929	900				
Educational level	Between group	57.757	4	14.439	9.551	5.63	.000
	Within group	1354.554	896	1.512			
Total		1412.311	900				
Sex of respondents	Between group	4.608	4	1.152	4.678	5.63	.001
	Within group	222.635	896	0.246			
Total		225.243	900				
Religion	Between group	0.819	4	0.205	1.499	5.63	.200
	Within group	122.346	896	0.137			
Total		123.165	900				
Occupation	Between group	72.047	4	18.012	8.807	5.63	.000
	Within group	1832.441	896	2.045			
Total		1904.488	900				
Income	Between group	155.388	4	38.847	14.676	5.63	.000
	Within group	2371.740	896	2.647			
Total		2527.128	900				

*Source*: Author's field survey (2009).

*Note*: Significant at *p* < .05.

In the same vein, the relationship between educational level, sex and occupation and perception of HIV/AIDS were found to be significant at *p* < .05 level of significance.

However, using ANOVA, Table 6 also indicated that there was no significant relationship between religious affiliation of respondents and perception of HIV/AIDS as the *F*^−tab^ for this variable was greater than *F*^−val^ and *p* > .05 which was an indication of no significant relationship between religious affiliation and perception of HIV/AIDS.

Test of Hypothesis 3: H_0_

‘There is no significant relationship between socio-economic characteristics (age, sex, level of education, marital status, religion, and occupation) of respondents and their reactions if a relative tests positive for HIV/AIDS.' To test this hypothesis, data collected on socio-economic characteristics of respondents and their reaction if a relative tests positive for HIV/AIDS were subjected to One-way Analysis of Variance (ANOVA).

The descriptive analysis of one-way ANOVA on the relationship between socio-economic characteristics of respondents and their reactions is shown in Table 7, if a relative tests positive for HIV/AIDS in Anambra, Nigeria at *p* < .05 level of significance. The result of the analysis for marital status showed that the Mean Squares between groups and within groups were 1.364 and 0.942, respectively (Msbg = 1.364, Mswg = 0.942). These yielded the *F*^−val^ of 1.447 and *F*^−tab^ of 8.53 which was not significant at *p* < .05 level of significance (*F*^−val^ = 1.447, *p* > .05). Therefore, from the statistical analysis below, the null hypothesis (H_0_) was accepted. This meant that there was no significant relationship between marital status and reaction if a relative tests positive for HIV/AIDS.

**Table 7.** Descriptive ANOVA on the relationship between socio-demographic characteristics of respondents and reactions if a relative tests positive for HIV/AIDS ANOVA.

Socio-demographic characteristics	Relationship between socio-demographic characteristics and reaction if relative tests positive for HIV/AIDS						
		Sum of squares	df	Mean square	*F*^−val^	*F*^−tab^	*p* < .05 Sig.
Marital status	Between group	4.091	3	1.364	1.447	8.53	.228
	Within group	848.992	901	0.942			
Total		853.083	904				
Educational level	Between group	90.659	3	30.220	20.505	8.53	.000
	Within group	1327.860	901	1.474			
Total		1418.519	904				
Sex of respondents	Between group	8.303	3	2.768	11.441	8.53	.000
	Within group	217.947	901	0.134			
Total		226.250	904				
Religion	Between group	2.408	3	0.803	5.988	8.53	.000
	Within group	120.778	901	0.134			
Total		123.187	904				
Occupation	Between group	28.718	3	9.573	4.578	8.53	.003
	Within group	1883.883	901	2.091			
Total		1912.601	904				
Income	Between group	117.169	3	39.056	14.595	8.53	.000
	Within group	2411.001	901	2.676			
Total		2528.170	904				

*Source*: Author's field survey (2009).

*Note*: Significant at *p* < .05.

However, ANOVA on the relationship between educational level and reaction is a relative tests positive for HIV/AIDS was significant at *p* < .05 level of significance with the mean squares between groups and within groups of 30.220 and 1.474, respectively (Msbg = 30.220, Mswg = 1.474). These yielded the *F*^−val^ of 20.505 and *F*^−tab^ of 8.53 which was significant at *p* < .05 level of significance (*F*^−val^ = 20.505, *p* < .05).

In the same vein, the relationship between sex, occupation and income and reaction of respondents if a relative tests positive for HIV/AIDS were found to be significant at *p* < .05 level of significance.

The ANOVA indicated a significant relationship between sex and perception of HIV/AIDS. Therefore, in order to know which sex has more positive perception of HIV/AIDS, *t*-test was conducted. Table 8 showed that females had a mean of 2.44 (with a standard deviation of 1.309) while males had a mean of 2.38 (with a standard deviation of 1.407) which meant that female had a greater mean than males which was an indication that females had more positive perception of HIV/AIDS than males.

**Table 8.** *t*-Test analysis to show between male and female, the group that has more positive perception of HIV/AIDS.

Sex	*N*	Mean	SD
*Respondents' perception of HIV/AIDS*			
Male	453	2.38	1.407
Female	448	2.44	1.309

## Discussion of findings

The socio-demographic characteristics of the respondents showed that a majority were secondary school graduates meaning that many of them were not highly educated which had serious implications for their perception of HIV/AIDS. The low level of divorce and separation in the area can be attributed to the marriage pattern in the area in which the marriage relationship is between two families and there is no room for divorce even after the death of a spouse.

Table 3 showed that a majority of the respondents saw the disease as a punishment from God. This finding corroborates Olubuloye *et al.* (1993) in a Nigerian study of the clergy's view of HIV/AIDS in which they found that a majority of the clergy saw the disease as a divine punishment from God. Perception accounts for the attitudes towards PLWHA and their families. This perception is as a result of the definition of HIV/AIDS corroborating the study's theoretical position that the definition of the situation determines the action of the people. These findings could be attributed to the culture of the people because culturally, the people define strange illnesses (illnesses without cure) as a punishment from God confirming Bleek's (1981) assertion that people attribute unknown illnesses to wrongs done to the gods by the sick person, which had attracted punishment in the form of a disease. It also corroborates Orubuloye *et al.* (1993) and Aniebue (2006) in the study of the Nigerian clergy's perception of HIV/AIDS in which they found that a majority defined HIV/AIDS as a punishment from God.

Also, many of the respondents defined HIV/AIDS as a disease that afflict immoral people. This perception could be attributed to the fact that many of the respondents saw sexual intercourse as the only way through which HIV/AIDS could be contracted. This is because promiscuity is frowned at in the study area and as such, there is no pity for a person suffering from any disease that is sexually transmitted in nature. This lack of pity is also part of the negative perception of the disease. This corroborates Caldwell *et al.* (1999) and Alubo *et al.* (2002) in a study in Benue State, Nigeria in which they found that there was no pity for PLWHA and that the respondents said that it is the person that eats pepper that should feel the bitterness. This means that PLWHA should bear the consequences of their action. According to Bleek (1981), it is within this context that people's negative perception to HIV/AIDS should be viewed. This perception was also one of the major sources of stigmatization of PLWHA. This perception was equally influenced by the level of knowledge of HIV/AIDS of the people because generally, the more people know about HIV/AIDS, the less they would see it as a disease of immoral people.

The study indicated that many of the respondents saw HIV/AIDS as real, but their level of knowledge of the disease was quite shallow considering that many of them do not feel that everybody was at risk and many also indicated that HIV is contracted through shaking of hands and some even indicated that they were using medicine to protect themselves. This signifies a low level of knowledge which influences the perception of the disease. This corroborates Ibrahim *et al.* (2007) and UNAIDS (2003) that there was a general low level of knowledge among the members of the public. For instance, a person that feels that HIV is transmitted through shaking of hands would definitely see PLWHA as outcasts who should not mingle with the public at all. The cause of this low level of knowledge could be as a result of a low level of education in the area. It could also be as a result of absence of government HIV- intervention programmes in the area.

Furthermore, the result of the tested hypotheses showed that only marital status was significant for the knowledge of HIV/AIDS. Others socio-demographic variables were not significant which was an indication that a person's socio-demographic status does not influence knowledge of HIV/AIDS in the study area. This corroborated Kaiser (2006) that at least 46% adult Americans hold a misconception. Also, the result of Hypothesis 2 showed that marital status, educational level, income, sex and occupation of the respondents all had significant relationships with the perception of HIV/AIDS. The reason for the influence of marital status could result in the fact that in the study area, the marriage bond is very strong. Also, the influence of sex could be as a result of the fact that women have always been very sympathetic to sick relations. Furthermore, the educational level influences perception of HIV/AIDS which could result in the fact that education enables people to have greater awareness about HIV/AIDS. The influence of income in the perception of HIV/AIDS could be because people with higher income levels were likely to be people with a higher level of education, which helped them to view the disease differently.

The result of Hypothesis 3 showed that the relationship between marital status and respondents' reaction if a relative tested positive for HIV was not significant. This could be because the blood relationship is very strong in these areas, which implies that no matter a person's marital status, he/she is more likely to care for a sick relative. Apart from marital status, all the other variables (income, sex, education, occupation, and religion) had a significant relationship with respondents’ reactions if a relative tested positive for HIV/AIDS. The reason for the influence of income on people's reaction to a relative's HIV positive status could be because poverty has a way of making people to run away from responsibility because a poor person may not have anything to offer a sick relation. This inability to afford anything may make the person to react negatively to a relative with HIV/AIDS. In the same vein, religion's influence on people's reaction to a relative's HIV positive status could result in the fact that religious affiliation calls for one to be easily sympathetic to the plight of others.

Furthermore, females were found to have a more positive perception of HIV/AIDS than males. This could result in the fact that females are usually more sympathetic to people who are suffering. Moreover, in the study area, every female views every child in the community as her child and this is also exhibited during illness.

## Implications for health workers in the area

Health workers have to implement a number of community awareness programmes to make PLWHIV and their families more acceptable to their communities and to disabuse the minds of the people of their negative perception of the disease. Therefore, a cultural and family approach is needed in HIV/AIDS prevention and care and in a bid to change the negative perception of the disease in Africa. Behavioural analysis and intervention points of entry into a community should focus on culture rather than on individual behaviours. The first step that should be taken by health workers in the area should be to mount HIV/AIDS educational campaigns and target communities through their community leaders. The health workers should educate the communities on how HIV is contacted so as to disabuse the peoples' mind of their negative perceptions of HIV/AIDS. This knowledge would help families to bring PLWHIV/AIDS to hospitals instead of confining them at home. Also, because it is difficult to reach PLHIV in the study area, health workers should find a way of using village informants to reach PLHIV and their families. Furthermore, the implication for prevention efforts is that families should be educated on the routes of contact and how to avoid being infected. This would help in the change of perception and attitudes generally.

## Conclusion and recommendations

In conclusion, the study found a negative perception and a low level of knowledge among the Igbo. The respondents saw HIV/AIDS as a punishment from God and as a disease of immoral people and this was a product of the people's cultural and religious beliefs. These perceptions influenced how PLHIV in the area were treated. It also influenced the behaviour of infected persons. Seeing the disease as a punishment from God and disease of immoral people had the serious implications of making the people not to have any form of pity for PLHIV. This also meant that PLHIV in the area hardly get help. It, therefore, followed that PLHIV in the study area would degenerate quickly from HIV to AIDS as they did not have much support. Furthermore, the fact that marital status and other socio-economic characteristics of the respondents influenced perception and reaction if a relation tests positive for HIV/AIDS was an indication of a uniform perception which meant that the people reacts alike to the issue irrespective of their social status. This was a sign that changing this negative perception and reducing stigma was likely to be a difficult task which would constitute a hindrance towards the containment of the pandemic.

It was, therefore, recommended that more awareness campaigns should be done in the study area to enlighten the people on HIV/AIDS and how it could be contracted or transmitted. This would increase their level of knowledge of the disease and would change this negative perception.
